# Cognitive Function in Acquired Bilateral Vestibulopathy: A Cross-Sectional Study on Cognition, Hearing, and Vestibular Loss

**DOI:** 10.3389/fnins.2019.00340

**Published:** 2019-04-24

**Authors:** Bieke Dobbels, Griet Mertens, Annick Gilles, Annes Claes, Julie Moyaert, Raymond van de Berg, Paul Van de Heyning, Olivier Vanderveken, Vincent Van Rompaey

**Affiliations:** ^1^Faculty of Medicine and Health Sciences, University of Antwerp, Antwerp, Belgium; ^2^Department of Otorhinolaryngology and Head and Neck Surgery, Antwerp University Hospital, Edegem, Belgium; ^3^Division of Balance Disorders, Department of Otorhinolaryngology and Head and Neck Surgery, Maastricht University Medical Center, Maastricht, Netherlands; ^4^Faculty of Physics, Tomsk State University, Tomsk, Russia

**Keywords:** bilateral vestibulopathy, hearing loss, sensorineural, COCH protein, human, causality, cognition

## Abstract

**Background:**

Several studies have demonstrated cognitive deficits in patients with bilateral vestibulopathy (BVP). So far, little attention has been paid to the hearing status of vestibular patients when evaluating their cognition. Given the well-established link between sensorineural hearing loss (SNHL) and cognitive decline and the high prevalence of SNHL in BVP patients, it is therefore uncertain if the cognitive deficits in BVP patients are solely due to their vestibular loss or might be, partially, explained by a concomitant SNHL.

**Objective:**

To evaluate the link between cognition, hearing, and vestibular loss in BVP patients.

**Design:**

Prospective cross-sectional analysis of cognitive performance in patients with BVP and control participants without vestibular loss. Both groups included subjects with a variety of hearing (dys)function. Cognition was assessed by means of the Repeatable Battery for the Assessment of Neuropsychological Status for Hearing Impaired Individuals (RBANS-H).

**Results:**

Sixty-four BVP patients were evaluated and compared with 83 control participants. For each subscale and the totale RBANS-H scale a multiple linear regression model was fitted with the following variables: vestibular loss, hearing loss, age, gender, and education. Hearing loss seemed to be associated with worse outcome on the total RBANS-H scale and subscales immediate memory and language. Vestibular loss, on the other hand, was linked to worse performance on the attention subscale of the RBANS-H. Furthermore, we did not observe a correlation between saccular function and cognition.

**Conclusion:**

This study has found general cognitive deficits in a large sample size of BVP patients. Multiple linear regression models revealed that both vestibular and hearing dysfunction were associated with different subscales of the cognitive test battery, the RBANS-H. Whereas hearing loss was associated with worse performance on total RBANS-H score, immediate memory and language, vestibular loss was observed to negatively affect attention performance.

## Introduction

The vestibular apparatus, which includes the three semicircular canals and two otolith organs, is known to be crucial in maintaining balance and gaze stabilization. However, a growing body of literature recognizes that the role of the vestibular system goes far beyond these primitive reflexes. Over the past decades, numerous animal studies have repeatedly demonstrated spatial cognitive deficits in rodents with vestibular lesions (Wallace et al., 2002; Zheng et al., 2007, 2009a,b, 2012a,b, 2013; Baek et al., 2010). These findings have prompted researchers to evaluate whether human patients with vestibular lesions also suffer from cognitive dysfunction. Because of their bilateral loss of peripheral vestibular input, patients with bilateral vestibulopathy (BVP) are of specific interest to investigate the cognitive repercussions of vestibular loss. Numerous studies have demonstrated spatial cognitive deficits in BVP patients (Brandt et al., 2005; Grabherr et al., 2011; Kremmyda et al., 2016; Popp et al., 2017; Xie et al., 2017; Wei et al., 2018). Besides spatial cognitive impairment, more recent studies also show evidence of general cognitive deficits in BVP patients (Bessot et al., 2012; Popp et al., 2017; Wei et al., 2018). For instance, Popp et al. showed a statistically significant impairment of executive function, visuospatial abilities, attention and short-term memory in BVP patients (Popp et al., 2017). These findings are supported by the recent findings of Wei and colleagues, who examines executive function and attention in patients with Alzheimer’s disease (AD) and mild cognitive impairment (MCI). In this study, subjects with bilateral absent saccular function performed significantly worse compared to peers with normal saccular function on the Trial Making Test-B (Wei et al., 2018).

The growing evidence of cognitive impairment in BVP patients has led to speculation on a causal relationship between peripheral vestibular loss and dementia (Previc, 2013; Harun et al., 2016). A recent cross-sectional study has found an increased prevalence of AD in individuals with bilateral absent saccular function (Harun et al., 2016). Up to now, convincing evidence to recognize vestibular loss as an independent risk-factor for dementia is lacking.

The pathomechanism that underpins the cognitive deficits in subjects with vestibular loss is not fully understood. Substantial research suggest that the extensive vestibular-cortical network forms the basis of this relationship (Smith and Zheng, 2013; Hitier et al., 2014; Smith, 2017). The vestibular system, more than any other sensory system, has widespread projections to many cortical areas, including those involved in autonomic functions, sleep, emotions, and cognition. As such, the spatial cognitive impairment in BVP patients seems to be related to an atrophy of the hippocampal formation, i.e., the hippocampus and parahippocampus, which is known to play a key role in spatial memory and navigation (Zheng et al., 2013; Hitier et al., 2014; Smith, 2017). Studies comparing brain volumes on MRI imaging using voxel-based morphometry, indeed revealed smaller hippocampal formation volumes in BVP patients compared with matched controls (Brandt et al., 2005; Gottlich et al., 2016; Kremmyda et al., 2016). Likewise, patients with Menière’s disease were observed to have hippocampal atrophy compared to healthy controls. Moreover, their hippocampal volume was significantly correlated with the severity of vestibular loss (*p* < 0.05) and hearing dysfunction (*p* < 0.001) (Seo et al., 2016).

However, probably related to the wide cortical vestibular network, vestibular patients are prone to also develop anxiety, depression, and sleep disturbances (van de Berg et al., 2015; Kremmyda et al., 2016; Smith, 2017; Lucieer et al., 2018; Smith et al., 2018). In addition, some of peripheral vestibular diseases are frequently associated with sensorineural hearing loss (SNHL), e.g., Menière’s disease, BVP, ototoxicity, … Both psychiatric comorbidities and hearing loss are known to negatively influence cognition themselves (Livingston et al., 2017). Multiple cohort (Lin et al., 2013; Gurgel et al., 2014) and cross-sectional studies (Lin, 2011; Lin et al., 2011; Quaranta et al., 2014; Loughrey et al., 2018) have demonstrated that SNHL is an independent risk factor for cognitive decline and even dementia. Therefore, the question is raised whether the observed cognitive deficits in vestibular patients are a result of these other comorbidities or whether they can be independently attributed to vestibular loss (Smith, 2017; Dobbels et al., 2018; Smith et al., 2018).

Recently, Smith and colleagues demonstrated short-term memory deficits in patients with a variety of vestibular syndromes, independently of psychiatric disorders, fatigue and sleep disturbances (Smith et al., 2018).

Yet, studies investigating cognition in vestibular patients thereby taking their hearing status into account, are limited. For example, Semenov and colleagues demonstrated cognitive deficits in 757 subjects with vestibular dysfunction, independently of SNHL. However, study participants did not receive laboratory vestibular testing to reassure peripheral vestibular loss. Moreover, only a subset of patients received auditory assessment (Semenov et al., 2016). A recent systematic review pointed out that none of the studies investigating cognition in BVP patients, diagnosed by means of calorics, rotatory chair test, and/or (video) head impulse test as suggested by the BVP diagnostic criteria of the Bárány Society, corrected for SNHL. Nonetheless, prevalence of SNHL in BVP patients range from 31 to 44% (Zingler et al., 2007; Lucieer et al., 2016).

Therefore, it is uncertain whether the cognitive impairment demonstrated in BVP patients could be solely attributed to the loss of vestibular input as suggested in previous studies. These cognitive deficits in BVP patients might be partially caused by a concomitant hearing loss.

In the light of the above uncertainty, this study specifically aims to evaluate the link between cognition, vestibular and hearing dysfunction in BVP patients with established vestibular loss by laboratory vestibular testing. Therefore, the general cognitive performance of BVP patients was compared to controls without vestibular loss and a variety of hearing (dys)function. To assess cognitive function, an instrument specifically designed to exclude any potential bias from hearing loss was used: the Repeatable Battery for the Assessment of Neuropsychological Status for Hearing Impaired Individuals (RBANS-H). This test battery assesses immediate and delayed memory, attention, language and visuospatial abilities.

## Materials and Methods

### Study Design

This was a single-center, prospective, cross-sectional study recruiting from October 2017 until August 2018 at the Antwerp University Hospital. BVP patients were assessed during two separate visits of approximately 1.5–2 h by two ICH-GCP-accredited clinical researchers. Cognitive assessment was always performed at the start of each visit – by means of the Repeatable Battery for the Assessment of Neuropsychological Status for Hearing Impaired Individuals (RBANS-H) – in order to avoid loss of attention due to fatigue.

### Study Participants

#### BVP Patients

Patients with a previous tentative diagnosis of BVP were recruited from the patient’s database at the Otorhinolaryngology, Head and Neck surgery department at the Antwerp University Hospital, Belgium. All patients who accepted enrolment in this study, received new neuro-otological testing on site. The evaluation of the lower frequencies function of the lateral semi-circular canals was performed by electronystagmography (ENG) with bithermal caloric tests and rotatory chair test (Nystagliner Toennies, Germany). At our clinic, rotatory chair tests are performed using sinusoidal rotation (0.05 Hz) with a peak velocity of 60°/sec. Further detailed methodology and normative data had been previously described (Van der Stappen et al., 2000). In the first 35 patients, caloric irrigation was performed with water. Due to change in local patient safety guidelines, caloric insufflation in the other patients had to be performed with air: warm (47°C) and cold (26°C) air for 30 s. High-frequency function of all six semicircular canals was measured by the video head impulse test (vHIT). Angular head velocity was determined by three mini-gyroscopes, eye velocity by means of an infrared camera recording the right eye, all incorporated in commercially available vHIT goggles (Otometrics, Taastrup, Denmark). Vestibulo-ocular reflex (VOR) gain was defined as the ratio of the area under the eye velocity curve to the head velocity curve from the impulse onset until the head velocity was again 0 (Macdougall et al., 2013). According to the recently established Bárány society criteria (Strupp et al., 2017), BVP diagnosis can be made based upon a bilaterally reduced function of the lateral semi-circular canals. Additionally, we evaluated saccular function by performing c-VEMP testing. Details on the procedure have been published previously (Colebatch et al., 1994; Vanspauwen et al., 2011). In brief, a patient’s saccular function is quantified by the response of the ipsilateral sternocleidomastoid muscle to air-conducted 500 Hz tone bursts delivered monoaurally via insert phones. Recordings were made with an auditory evoked potential system equipped with electromyographic software (Neuro-Audio, Difra, Belgium), with self-adhesives electrodes (Blue sensor, Ambu, Denmark). The presence of a typically biphasic shape, with a positive peak after 13 ms (p13) and a negative peak after 23 ms (n23), was evaluated. When no p13n23 wave was seen above 100 dB acoustic clicks, a patient was considered to have an absent cVEMP response.

Inclusion criteria for BVP patients were:

(1) Bilaterally reduced vestibular function, as defined by the Bárány Society Criteria for BVP (Strupp et al., 2017):
  - horizontal angular VOR gain < 0.6 measured by the vHIT, and/or
  - reduced caloric response (sum of bithermal, 30 and 44°, maximum peak slow phase velocity (SPV) on each side < 6°/sec), and/or
  - reduced horizontal angular VOR gain < 0.1 upon sinusoidal stimulation on a rotatory chair
(2) Disease duration of BVP > 6 months, in order to exclude acute pathology with possible remaining vertigo spells. Moreover, in the case of a vestibular neuritis recovery can occur in the first 6 months.
(3) Subjects might suffer from a post-lingual hearing loss.

#### Control Participants (CPs)

Control participants were recruited on the one hand, for subjects with hearing loss, from the hospital patient’s database and on the other hand, for subjects without expected hearing loss, by means of the population registries at the local city councils in southern Antwerp (Belgium), by advertisements in the hospital and by approaching friends, family, and colleagues. Only subjects with no history of vertigo and scores < 5 on the Dizziness Handicap Inventory (DHI) were considered for enrolment in the study. After a screening phase, in which medical history was questioned and the DHI administered, hearing performance was examined. If participants suffered from SNHL, rotatory chair test with electronystagmographic registration of the vestibulo-ocular reflex was performed to exclude severe vestibular dysfunction. Only subjects with a gain > 0.3 were enrolled as controls in the study. This cut-off value was chosen in compliance with the current criteria for presbyvestibulopathy, which are in development by the Bárány Society (Agrawal et al., 2018). In these criteria, a gain lower than 0.3 upon sinusoidal stimulation on a rotatory chair is considered pathological.

For both BVP patients and CP the following additional inclusion criteria were applied: (1) Age ≥ 18 years; (2) Fluency in Dutch; (3) No history of neurological diseases (e.g., dementia, Parkinson’s disease, cerebrovascular accident, etc.); (4) Absence of clinical signs indicating dementia or MCI.

Education of all participants was classified as primary school, lower secondary school, upper secondary school and college/university.

### Cognitive Assessment

The assessment of cognition was based on a validated neuropsychological test battery, designed by Randolph: the Repeatable Battery for the Assessment of Neuropsychological Status (RBANS) (Randolph et al., 1998; Randolph, 2012). Advantages of this test battery include the comprehensive insight on general cognitive performance, the relatively short administering time (30–40 min), good sensitivity, the established test–retest reliability and its age-corrected normative data (Duff et al., 2005; Randolph, 2012).

In the RBANS-H, modifications are made to create a test battery suitable for hearing impaired subjects (Claes et al., 2016). In addition to the RBANS, in which all instructions are given orally, written instructions through PowerPoint presentation are provided (Claes et al., 2016). Regarding the inclusion of participants with SNHL, the additional written instructions are necessary to test our study population. With only oral instructions, patients with SNHL are at a possible disadvantage of not hearing correctly the instructions and thus obtain lower scores compared with normal hearing peers.

Similar to the RBANS, the RBANS-H is a complete cognitive test battery consisting of 12 subtests evaluating five cognitive domains: immediate memory, delayed memory, attention, language and visuospatial abilities (Table 1).

**Table 1:** The five cognitive domains and 12 subtests of the RBANS.

Immediate memory	Delayed memory	Visuospatial	Language	Attention
List learning	List recall	Figure copy	Picture naming	Digit span
Story memory	List recognition	Line orientation	Semantic fluency	Coding
	Story recall			
	Figure recall			

As considered of special interest, the tasks evaluating visuospatial cognition will be discussed briefly. In the task ‘Figure Copy,’ participants are shown a complex geometric figure on a screen. While remaining visually available, participants are asked to copy the figure on a paper with special attention to the correctness of each element and its relative location to the rest of the figure. In the second subtest, Line orientation, 13 identical lines radiating out from a single point and forming half of a circular pattern, are shown. Below this pattern of numbered lines (1–13), two identical lines that match two of the lines from the original pattern are displayed. The participant is asked to give the numbers of the lines in the original pattern to which the two lines correspond.

All subtests are scored separately to generate the index score of the corresponding cognitive domain, and total score RBANS-H score. All index and total scores can be converted to age-appropriate scores with a normal distribution with a mean of 100 and a standard deviation of 15.

### Hearing Assessment

To correct cognitive outcome measures for the hearing status of the enrolled participants, a speech audiometry in noise was performed. In order to correspond as much as possible to their daily life hearing performance, participants were asked to use their habitual hearing aids if they had any. Assessment was conducted in free field in an audiometric soundproof booth using the Leuven Intelligibility Sentences Test (LIST) (van Wieringen and Wouters, 2008). This speech material consists of several lists of 10 sentences. An adaptive procedure was used to determine the speech reception threshold (SRT). The level of the speech-weighted noise was fixed at 65 dB SPL and the intensity level of the sentences varied in steps of 2 dB adaptively in a one-down, one-up procedure according to participant’s response. The SRT was ascertained based on the level of the last six sentences of two lists, including an imaginary 11th sentence. A lower SRT indicates better speech in noise perception and thus better hearing function. The average SRT in an normal hearing population is −7.8 ± 1.17 dB speech-to-noise ratio (van Wieringen and Wouters, 2008).

### Data Collection and Statistics

Data were stored in OpenClinica LLC (Waltham, MA, United States), an online database for electronic data registration and data management developed for clinical research. Data were stored with a password secured access to the online database. We used IBMS SPSS Statistics 24 (IBM; Armonk, NY, United States) for the statistical analyses. Demographic data were analyzed with *t*-test (continuous data) and chi-squared test (categorical data), or the appropriate non-parametric tests. To adjust for confounders in the comparison of cognitive performance between BVP patients and CP, a multiple linear regression model was fitted. The RBANS-H score, either the total scale or the subscales, was entered as dependent variable. Group, SNHL (SRT in noise), age, gender, and education were entered as covariates in all models. These models evaluate whether BVP patients have worse cognitive function than CP when correcting for hearing dysfunction and other confounders. The same models can test if the effect of SNHL on the RBANS-H (sub)scores is significant, when adjusting for vestibular loss and other confounders. Multicollinearity was checked by means of variance inflation factors. Normality of the residuals were checked with QQ-plots. Homoscedasticity, independence of error and linearity assumptions were controlled by means of residuals plots.

Finally, a linear regression model was fitted to assess the link between saccular function and cognition in the BVP group. Both, the amplitude of the p13n23 wave and the cVEMP response (unilateral absent, bilateral absent, or bilateral present response) were entered as dependent variable. Again, these two models were adjusted for SNHL (SRT in noise), age, sex, and education.

## Results

### Study Population

The database consisted of 234 patients with a tentative diagnosis of bilateral vestibulopathy. Of these patients, 127 patients declined to participate in the study, 34 patients did not meet the Bárány Society criteria for BVP based on their vestibular test results, three patients did not speak Dutch and two patients died before the start of the study.

Finally, sixty-four BVP patients with a mean age of 59 ± 14.3 years met the study inclusion criteria; 59% of them were male. Eighty-three CP with a mean age of 68.4 ± 10.5 years, were enrolled in the study; 52% of them were male (Table 2). There was no statistically significant difference between BVP patients and CP concerning sex, education level and hearing performance (SRT in noise). CP were significantly older than BVP patients.

**Table 2:** Demographic characteristics and hearing status of study population.

	BVP patients	Control participants	
	*n* = 64	*n* = 83	
Age (mean, SD)	59 (14.3)	68.4 (10.5)	*p* < 0.05
Sex (*n*, %)			*p* = 0.4
Male	38 (59)	43 (52)	
Female	26 (41)	40 (48)	
Education (*n*, %)			*p* = 0.1
Less than primary school	1 (1.8)	0	
Primary school	2 (3.6)	12 (14.5)	
Lower secondary school	11 (19.6)	10 (12)	
Upper secondary school	20 (35.7)	23 (27.7)	
College/University	22 (39.3)	38 (45.8)	
Hearing performance			
Speech reception threshold in noise (mean, SD in dB)	+0.65 (± 6.5)	−0.27 (± 7.4)	*p* = 0.4
Conventional hearing aid (n)	12	12	
Other hearing aid: CI, BAHA (n)	19	1	

*BVP, bilateral vestibulopathy; CI, cochlear implant; BAHA, bone anchored hearing aid.*

**FIGURE 1:**
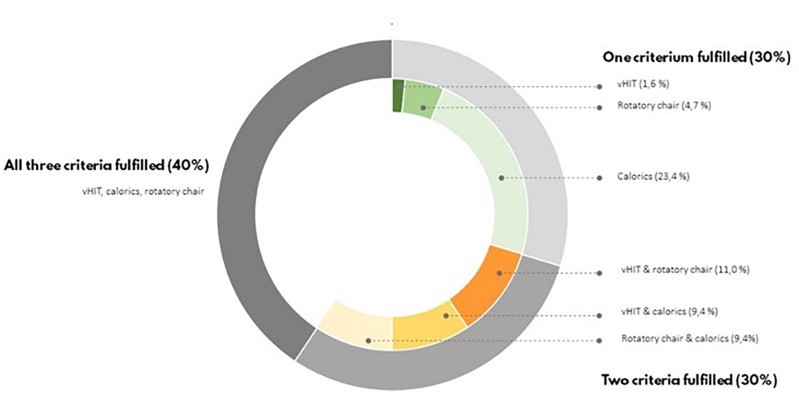
According to the Barany Criteria, bilateral vestibular loss is defined by a hypofunction measured by means of caloric irrigation, rotatory chair test, or vHIT. In this figure, the percentages of BVP who fulfilled all these criteria or less are presented.

To be enrolled as BVP patient, the Bárány Society criteria needed to be fulfilled (Strupp et al., 2017). Forty percent of BVP patients met all Bárány society criteria: a bilateral reduced response on caloric testing, rotatory chair test and vHIT. In 30% of BVP patients two out of three Bárány society criteria were fulfilled, and in the remaining 30% of the BVP patients there was only found a vestibular hypofunction in one of the three vestibular tests (Figure 1).

**FIGURE 2:**
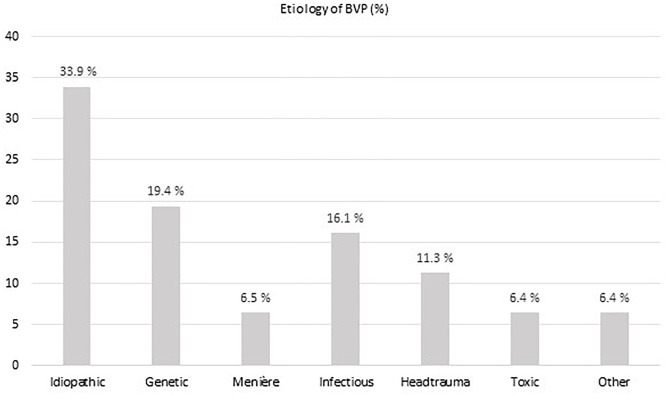
The different etiologies of bilateral vestibulopathy (BVP) in the study cohort.

An underlying cause of vestibular loss could not be identified in 33.9% of BVP patients. With a prevalence of nearly 20%, a mutation in the COCH gene causing DFNA9, was the most frequent underlying non-idiopathic etiology in our BVP cohort. In 16% of BVP patients, an infectious cause was found (e.g., meningitis, neuritis, Lyme disease). Menière’s disease and head trauma accounted for respectively 6 and 11% of BVP causes. In four BVP patients an ototoxic cause was suspected (3 aminoglycosides antibiotics and 1 chemotherapy, not further specified) (Figure 2).

Among the BVP patients, the mean SRT during speech in noise was +0.65 (± 6.5) dB SNR and ranged from −7 to +20 dB SNR. This was not significantly different from control participants (mean SRT during speech in noise −0.27 ( ± 7.4); range −7 to +20 dB SNR).

### The Effect of Vestibular Loss and Hearing Loss on Cognitive Performance

All 64 BVP patients completed the RBANS-H except from five participants: four refused any cognitive assessment; one participant witnessed the RBANS-H test procedure in his father and was thus excluded because of a potential practice effect. Thus, in total 59 BVP patients were included for statistical analysis concerning RBANS-H results. We did not observe a statistically significant difference on total RBANS-H score or any RBANS-H subscales between BVP patients diagnosed by caloric irrigation with water, compared with those diagnosed by insufflation with air.

**FIGURE 3:**
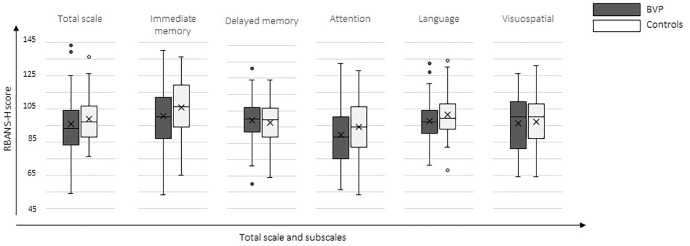
Cognitive outcome measures. Boxplots of the RBANS-H total and index scores are shown for the CP group (control participants) and BVP group (bilateral vestibulopathy).

Results of the RBANS-H total score and index-scores are presented in Figure 3. Overall, BVP patients performed worse on the RBANS-H (mean total score in BVP patients 95.6 ± 17.2 versus 98.7 ± 13.1 in CP). Mean performance on Immediate Memory, Attention, Language, and Visuospatial was worse in BVP patients (see Table 3). Delayed Memory seemed to be slightly better in BVP patients compared to CP.

To evaluate the presence of a statistically significant association between vestibular loss, SNHL and cognition, a multiple linear regression model was conducted for each outcome measure (total RBANS-H scale and every subscale) (Table 3). All models were adjusted for SNHL (SRT in noise), age, sex, and education. As previously mentioned, the same model evaluates whether there is a statistically significant effect of group (BVP versus CP) and SNHL on the cognitive outcome measure. Model coefficients were designed in such way that a negative model coefficient always indicated poorer cognition in the BVP group. Similarly, a negative coefficient pointed at a negative impact of SNHL (higher SRT) on cognitive performance.

**Table 3:** Results of the Repeatable Battery for the Assessment of Neuropsychological Status for Hearing Impaired Individuals (RBANS-H) total and index scores in BVP patients and CP.

	Total RBANS-H scale		Immediate memory		Delayed memory		Attention		Language		Visuospatial	
**Mean performance**												
BVP patients: mean (SD)	95.6 (17.2)		100.4 (18.4)		99.1 (14.2)		89.1 (18.0)		97.7 (11.6)		96.3 (16.3)	
CP: mean (SD)	98.7 (13.1)		105.7 (15.9)		97.9 (12.4)		94.2 (16.1)		101.3 (12.1)		96.9 (14.2)	

**Linear regression model**	***p*-value**	**β**	***p*-value**	**β**	***p*-value**	**β**	***p*-value**	**β**	***p*-value**	**β**	***p*-value**	**β**

BVP versus CP	0.142	−3.7	0.07	−5.0	0.73	0.8	0.045	−5.9	0.083	−3.6	0.396	−2.4
Hearing status (SRT)	0.027	−0.4	0.009	−0.5	0.115	−0.3	0.104	−0.4	0.002	−0.5	0.492	0.1

*Linear regression analyses were conducted to assess the association between vestibular loss, hearing loss and cognition. All models adjusted for hearing loss (SRT in noise), age, sex, and education. Statistical significant p-values are shown in gray. BVP, bilateral vestibulopathy; CP, healthy controls; SRT, speech reception threshold; RBANS-H, The Repeatable Battery for the Assessment of Neuropsychological Status for Hearing Impaired Individuals.*

Regarding the link between vestibular loss and cognition, only a statistically significant difference was found between BVP patients and CP on the subscale Attention (mean score of 89.1 ± 18 in BVP patients, versus mean score of 94.2 ± 16.1 in CP, *p* = 0.045, β = −5.9). In contrast, hearing dysfunction, in both BVP and CP,was statistically significant associated with worse cognitive performance on the Total scale of the RBANS-H and on the subscales Immediate Memory and Language. More specifically, one dB lower SRT in noise was associated with a 0.4 lower Total scale (*p* = 0.027), a 0.6 lower Immediate Memory index score (*p* = 0.009) and a 0.5 lower Language score (*p* = 0.002).

### Saccular Function and Cognition

Only four BVP patients had a bilateral preserved saccular function. Twenty-seven patients had bilateral absent c-VEMP responses, and in 13 BVP patients a unilateral c-VEMP response was measured. Due to practical considerations or insufficient sternocleidomastoid muscle tension, c-VEMP responses could not be reliably measured in the remaining 20 patients. *Total RBANS-H score* was higher in patients with bilateral present saccular function (mean: 116 ± 9.8) than in patients with unilateral preserved saccular function (mean: 95.8 ± 17.5) and bilateral loss of saccular function (90.4 ± 15.6). However, with correction for SNHL, age, sex and education, this effect did not reach level of statistical significance (*p* = 0.253). Furthermore, no significant associations were found between the presence of a c-VEMP response (unilateral present, bilateral present or bilateral absent) and the five different *sub scales of the RBANS-H*. Finally, an association between cognitive performance (total RBANS-H scale, all subscales) and c-VEMP amplitude in the better ear was explored in a linear regression model for each outcome, all correcting for SNHL, age, gender and education (Table 4). Neither on the RBANS-H total scale, nor on any of the index scores, a significant correlation was revealed between the p13-n23 wave amplitude and cognitive performance.

**Table 4:** The Repeatable Battery for the Assessment of Neuropsychological Status for Hearing Impaired Individuals (RBANS-H) total and index scores regarding c-VEMP response in BVP patients.

	Total scale	Immediate memory	Delayed memory	Attention	Language	Visuospatial
**Mean performance**						
Bilateral absent c-VEMP response: mean (SD)	90.4 (15.6)	96.0 (18.3)	96.9 (15.5)	85.7 (16.8)	94.2 (11.0)	91.2 (17.1)
Unilateral present c-VEMP response: mean (SD)	95.8 (17.5)	96.5 (16.3)	100.0 (9.4)	90.2 (21.5)	100.0 (13.5)	96.5 (11.0)
Bilateral present c-VEMP response: mean (SD)	116.0 (9.8)	124.5 (13.7)	110.3 (11.6)	101.5 (7.1)	107.8 (7.6)	112.8 (11.0)
**Linear regression model in 3 VEMP groups** *(bilateral absent response/unilateral present response/bilateral present response)*						
*p*-value	0.4	0.06	0.8	0.5	0.4	0.1
**Linear regression model with amplitude of c-VEMP of the better ear**						
*p*-value	0.6	0.3	1	0.8	0.5	0.6

*c-VEMP, cervical vestibular evoked myogenic potentials. All linear regression models adjusted for hearing (speech reception threshold in noise), age, sex, and education.*

## Discussion

Despite the extensive research demonstrating cognitive impairment in animal and human subjects suffering from vestibular lesions, it remains a matter of debate to which extent these cognitive deficits are solely dependent on their vestibular loss. As such, BVP patients frequently suffer from psychiatric comorbidities and hearing loss, both known to negatively affect cognitive functioning. To the best of our knowledge, this is the first study evaluating cognition in a large cohort of 64 BVP patients with laboratory confirmed vestibular loss, that controls for the hearing (dys)function of the test subjects. The used cognitive test battery, the RBANS-H, is especially designed to exclude any bias from hearing loss. The BVP group and CP group both included subjects with a variety of hearing dysfunction.

### Unraveling the Relationship Between Hearing Loss, Vestibular Loss, and Cognition in BVP Patients

In general, BVP patients were observed to perform worse on the total RBANS-H scale and the following subscales: immediate memory, attention, language, and visuospatial. By means of a multiple linear regression model fitted for each outcome measure, it was evaluated if the worse outcome in BVP patients was significantly linked to vestibular loss (group) or hearing loss (SRT in noise), thereby additionally adjusting for age, gender and education. Hearing loss, in both BVP patients and CP, was observed to be associated with a worse score on the total RBANS-H scale and on the subscales immediate memory and language. More specifically, 5 dB higher SRT resulted on average in two points lower on the total RBANS-H scale, three points lower on immediate memory and 2.5 points lower on language. These effects can be considered rather small (Randolph, 2012). On the other hand, vestibular loss seemed to be associated with a worse performance on the attention tasks of the RBANS-H. BVP patients scored on average 6 points less on the attention sub score.

In other words, these results confirm the previously observed cognitive deficits in BVP patients. However, it seems that not only the vestibular loss of BVP patients, but also their hearing loss, contributes to their impaired cognition. Particularly, attention seemed to be associated with vestibular (dys)function in this study. Interestingly, next to spatial cognitive deficits, attentional deficits have indeed been observed most frequently in previous BVP cohort studies (Dobbels et al., 2018). However, also memory deficits have been previously described in BVP patients (Popp et al., 2017). Our results suggest that these deficits might be more correlated to the hearing loss of BVP patients than to their vestibular loss. Language function, which is in this study negatively influenced by hearing loss, has never been assessed before in BVP patients.

### Spatial Cognition in BVP Patients

Because numerous studies have observed spatial cognitive deficits in animals and humans with vestibular loss, it is of potential interest that our results could not confirm this finding in a relatively large sample size of BVP patients. Even without correction for hearing loss, no significant difference in Visuospatial performance was found between CP and BVP patients. This rather contradictory result might rely on the kind of tasks used to assess visuospatial abilities. Previous literature seem to be more focused on spatial memory and navigation than constructional cognition (e.g., copying a figure) (Jandl et al., 2015; Kremmyda et al., 2016; Brandt and Dieterich, 2017; Popp et al., 2017). It is reasonable that the figure coping and line orientation task are not sensitive enough to detect spatial cognitive deficits in BVP patients. Moreover, a big animal literature repeatedly linked spatial cognition to the vestibular system. Several of these studies attempted to control for the bias of SNHL by removing the tympanic membrane of the sham control animals. Although this is only considered a partial auditory control, researchers repeatedly found that rodents without vestibular lesions but with a removed tympanic membrane outperformed rodents with vestibular lesions on a variety of spatial cognitive tasks (Zheng et al., 2007, 2009a,b, 2012b; Baek et al., 2010; Smith and Zheng, 2013).

In human BVP patients, the virtual Morris water task have been shown to be sensitive enough to detect spatial cognitive deficits in (small sample sizes of) BVP patients (Brandt et al., 2005; Kremmyda et al., 2016). Further research should be undertaken to investigate if spatial memory and navigation is impaired in BVP patients, regardless of their hearing status., for example by using the virtual Morris water task (Hamilton et al., 2009).

### Hearing Loss in BVP Patients

Compared with literature, the prevalence of SNHL in our BVP cohort was relatively high: 85% BVP patients with abnormal hearing in at least one ear. This might lead to a more substantial effect of hearing loss on cognition in our BVP patients compared to other BVP study groups. Therefore, these results need to be interpreted with caution and must be reproduced by future research. A possible explanation for the high occurrence of hearing loss in our BVP cohort, might be the high prevalence of genetic causes of BVP, i.e., COCH mutations. This is an autosomal dominant disorder, causing progressive otovestibular decline starting at the 3rd to 5th life decade. As the burden of a genetic disease might not be underestimated, it is not excluded that cognitive evolution behaves differently in DFNA9 patients compared with BVP patients caused by other pathologies. According to a recent literature review, cognition has never been explored in DFNA9 patients (De Belder et al., 2017).

Nonetheless, our findings underpin the importance of adjusting for hearing when reporting on cognition in a vestibular patient group. On the one hand, it is necessary to correct for hearing when investigating cognition in patients with vestibular disorders. Since positive effects of hearing aids on cognition have been reported in literature (Amieva et al., 2015), we chose to adjust for hearing in a best aided situation with the SRT in noise. When relying on hearing in an unaided situation, the effect of hearing might be overestimated as it does not represent the daily life situation of a patient. On the other hand, the instrument of cognitive assessment needs to be adapted for hearing-impaired participants. Otherwise, hearing loss might negatively affect the cognitive outcomes, as the to-be-repeated or to-be-remembered items may not be well-perceived by the participant (Dupuis et al., 2015).

Given the negative association between vestibular loss and attention and the close anatomical link between the cochlea and the vestibular system, future research investigating cognition in patients with severe hearing loss should take, vice versa, the vestibular function of the study group into account.

### Otolith Function and Cognition

To the best of our knowledge, previous research investigating cognition in BVP patients has not corrected for hearing (Glasauer et al., 1994, 2002; Brandt et al., 2005; Grabherr et al., 2011; Bessot et al., 2012; Jandl et al., 2015; Kremmyda et al., 2016; Popp et al., 2017). In these studies a diagnosis of BVP was based on bilateral hypofunction of the lateral semicircular canal, which is equivalent to the Bárány society criteria applied in this study. As the Bárány society criteria were only recently established, cut-off values to determine vestibular hypofunction vary across the latter studies (Dobbels et al., 2018). Interestingly, few studies found an association of saccular function with cognition (Bigelow and Agrawal, 2015; Xie et al., 2017; Wei et al., 2018). Otolith function, measured by ocular or cervical vestibular evoked myogenic potentials, is not incorporated in the Bárány society criteria for BVP. Importantly, one of the studies investigating saccular function and cognition adjusted for hearing (Bigelow et al., 2015). In this study, patients with absent c-VEMP response were excluded. After correction for hearing loss, a significant link between c-VEMP amplitude in the better ear with cognition remained. Therefore, we additionally investigated a link between saccular function and cognition in our BVP cohort. First, the association between a present or absent c-VEMP and cognitive performance was explored. Second, the amplitude of the c-VEMP response in the better ear was used as value for saccular function in the analysis. However, in contrast with the study of Bigelow and colleagues, we did not detect a significant correlation between saccular function on cognition. This might be mediated by the differences between both study protocols. In our study, we only analyzed saccular function in the BVP cohort and not in CP. Literature about the contribution of the saccule to cognitive function is sparse. Future research investigating this link might be of particular interest.

### Limitations

Due to the establishment of new patient safety guidelines at the Antwerp University Hospital, the electronystagmography procedure was changed from caloric irrigation with water to air insufflation. In total, 35 BVP patients received caloric irrigation with water. As higher responses are obtained for water irrigation compared to air insufflation, the former group of 35 BVP patients might suffer from a more profound vestibular loss the latter group with air irrigation (Maes et al., 2007). Surprisingly, the former group patients performed better on all subscales and on total RBANS-H scores, although not at a statistically significant level. Furthermore, the group of CP was significantly older than the BVP group. This seemed an inevitable consequence of matching CP and BVP on their hearing status. SNHL prevalence is relatively high in young BVP patients, whereas young controls with SNHL but without vestibular loss, confirmed by rotatory chair testing, are sparse. Yet, all subscales and the total RBANS-H scale are age-corrected. Furthermore, the disadvantage of the significantly older control group was tried to minimize by entering age as a covariate in all statistical models. Nonetheless, the older CP group makes it more difficult to reveal significant cognitive deficits in the BVP group.

Finally, this the first study using the RBANS(-H) to assess cognition in vestibular patients. The RBANS-H was chosen because it is a comprehensive test evaluating nearly all cognitive subdomains, thereby providing age-corrected sub- and total scores. Furthermore, Claes and colleagues demonstrated its suitability to test cognition in patients with severe SNHL (Claes et al., 2016, 2018a,b). The RBANS is more sensitive compared to cognitive screening tests such as the Montreal Cognitive Assessment (MoCA) (Claes et al., 2016). Nonetheless, as the RBANS-H has not been compared to other test batteries in vestibular patients, its sensitivity to detect cognitive dysfunction in this population is unknown.

## Conclusion

The current study was designed to evaluate the relationship between cognition, hearing, and vestibular loss in BVP patients. Cognitive performance was compared between BVP patients and subjects without vestibular loss, by using the RBANS-H, a cognitive task adapted for hearing-impaired subjects. In both groups subjects with a variety of hearing (dys)function were included. On the one hand a significant negative association was observed between hearing loss and total RBANS-H score, immediate memory and language. On the other hand, vestibular loss was associated with worse performance on the attention subscale of the RBANS-H.

## Ethics Statement

The study was approved by the local ethics committee of the Antwerp University Hospital/University of Antwerp (Protocol No. 16/42/426) and informed consent was obtained in all study participants prior to the start of the study. The study was registered on ClinicalTrials.gov (NCT03690817).

## Author Contributions

BD conceived and designed the study, acquired, analyzed, and interpreted the data, and wrote the manuscript with input from all authors. GM and AG conceived and designed the study, analyzed and interpreted the data, and critically revised the manuscript for important intellectual content. JM acquired and stored the data. AC contributed to normative data cognitive assessment. RVdB critically revised the manuscript for important intellectual content. PVH and VVR conceived and designed the study, analyzed and interpreted the data, critically revised the manuscript for important intellectual content, and supervised the study. OV critically revised the manuscript for important intellectual content and supervised the study.

## Conflict of Interest Statement

The authors declare that the research was conducted in the absence of any commercial or financial relationships that could be construed as a potential conflict of interest.
